# Molecular Typing of *Leishmania* spp. Causing Tegumentary Leishmaniasis in Northeastern Italy, 2014–2020

**DOI:** 10.3390/pathogens13010019

**Published:** 2023-12-24

**Authors:** Tommaso Gritti, Elena Carra, Gert Van der Auwera, José Carlos Solana, Valeria Gaspari, Silvana Trincone, Margherita Ortalli, Alice Rabitti, Alessandro Reggiani, Gianluca Rugna, Stefania Varani, The Skin_Leish_RER Network

**Affiliations:** 1 Department of Medical and Surgical Sciences, University of Bologna, 40126 Bologna, Italy; tommaso.gritti2@unibo.it (T.G.); margherita.ortalli2@unibo.it (M.O.); stefania.varani@unibo.it (S.V.); 2 Istituto Zooprofilattico Sperimentale della Lombardia e dell’Emilia Romagna, 25124 Brescia, Italy; elena.carra@izsler.it (E.C.); alice.rabitti@izsler.it (A.R.);; 3 Institute of Tropical Medicine, 2180 Antwerp, Belgium; gvdauwera@itg.be; 4 Centro de Investigación Biomédica en Red de Enfermedades Infecciosas, Instituto de Salud Carlos III, 28022 Madrid, Spain; 5 WHO Collaborating Centre for Leishmaniasis, National Center for Microbiology, Instituto de Salud Carlos III, 28022 Majadahonda, Spain; 6 Unit of Dermatology, Head and Neck Department, IRCCS Azienda Ospedaliero-Universitaria di Bologna, 40126 Bologna, Italy; 7 Unit of Dermatology, Ospedale Bufalini, Azienda Unità Sanitaria Locale della Romagna, 47521 Cesena, Italy; silvana.trincone@auslromagna.it; 8 Unit of Microbiology, IRCCS Azienda Ospedaliero-Universitaria di Bologna, 40126 Bologna, Italy

**Keywords:** *Leishmania*, heat shock protein 70, tegumentary leishmaniasis, sequencing, whole genome sequencing

## Abstract

Tegumentary leishmaniasis (TL) is endemic but neglected in southern Europe. Therefore, this study aimed to analyze the *Leishmania* strains causing TL cases in northeastern Italy, where an upsurge of TL cases has been observed in the last decade. Sections from 109 formalin-fixed and paraffin-embedded (FFPE) biopsies of skin and mucosal tissues were collected from TL cases in the selected area. Two DNA targets were amplified and sequenced: the ribosomal internal transcribed spacer 1 (ITS1) and the heat-shock protein 70 gene (hsp70). An in silico analysis was also performed on 149 genomes belonging to the *Leishmania donovani* complex. A total of 88 out of 109 (80.7%) samples from 83 TL cases were successfully typed by ITS1 and/or hsp70. ITS1 analysis identified *L. infantum* in 67 cases (91.8%), while *L. major* (n = 4, 5.5%) and *L. tropica* (n = 2, 2.7%) were detected in the remaining cases that were categorized as imported. Further, the hsp70 typing of 75 autochthonous cases showed the presence of eight distinct sequence variants belonging to the *Leishmania donovani* complex, with high genetic variability when compared to known *L. infantum* populations. In conclusion, our findings show that peculiar *L. infantum* variants are emerging in the novel focus on TL in northeastern Italy.

## 1. Introduction

Leishmaniases are parasitic vector-borne diseases caused by protozoa of the genus *Leishmania*. Infection transmission is caused by the bites of phlebotomine sand flies [1]. The clinical spectrum of the disease can vary from systemic infection (visceral leishmaniasis, VL) to parasite replication in the skin and/or mucous membranes. The latter is defined as tegumentary leishmaniasis (TL) and includes cutaneous leishmaniasis (CL), mucosal leishmaniasis (ML), and mucocutaneous leishmaniasis (MCL) [1].

The most frequent clinical form of leishmaniasis is CL; 221,614 cases were reported worldwide to the WHO in 2021 [2]. Complicated CL cases can progress into mucosal disease, diffuse CL, or disseminated CL; this tendency mainly depends on the parasite species and/or the host immune response [3]. TL is endemic in the Mediterranean basin, including Italy, where it is found in the southern part of the peninsula, Sardinia, Sicily, and the Tyrrhenian littoral [4]. Nevertheless, the epidemiological dynamics of leishmaniasis are changing in this Mediterranean country, leading to a rise in canine and human cases in the northern regions. In this context, an upsurge in TL cases was recently observed in the Emilia-Romagna region (RER), in northeastern Italy; in fact, the yearly incidence rose from below 0.2/100,000 inhabitants in 2010–2012 to 0.54–0.98 cases/100,000 inhabitants in 2017–2020 [5,6].

*Leishmania infantum* is the parasitic species causing TL in southwestern Europe; the main clinical form caused by this species is uncomplicated CL, and the most frequent clinical presentation is a single papule, which progresses over weeks to months to form a nodule with a central crust [3,7]. ML lesions caused by *L. infantum* are not clearly reported to be associated with previous CL [7]; ML develops predominantly in the buccal area, the pharyngeal and laryngeal area, and the nose [8,9]. *L. infantum* can disseminate to the mucous membranes with a higher frequency than previously reported [5,7]. Other *Leishmania* species can cause TL in the Old World, including *L. major* and *L. tropica*, though these species typically do not cause mucosal lesions [7].

The increase in TL cases in northeastern Italy, along with a non-expected high rate of ML cases [5], requires the proper species and strain characterization of endemic and imported cases in this region. The identification of *Leishmania* species by molecular methods has replaced multi-locus enzyme electrophoresis (MLEE) in the last decade, with the advantage of not requiring parasite isolation in culture [3]; different molecular markers have also been used for species identification and strain typing, including malic enzyme, glucose-6-phosphate dehydrogenase, mini-exon, the 7SL RNA gene, the internal transcribed spacers (ITS)1 and 2 of the ribosomal DNA (rDNA), and the heat-shock protein gene (hsp)70 [10,11].

The current study was aimed at typing *Leishmania* in clinical samples obtained from human TL cases in northeastern Italy in order to analyze the parasitic species and strains causing autochthonous as well as imported cases. For these purposes, we selected two target sequences: 1. The ITS1 sequence of ribosomal DNA, which has been widely used for the direct detection and identification of Old World *Leishmania* species in clinical samples [12,13], and 2. the hsp70 gene coding sequence, which is present in several copies arranged in tandem repeated units with almost no variation described in the coding sequences of all copies [14]. Nevertheless, occasionally more than a single allele is evidenced by the presence of two nucleotides in the same sequence position. hsp70 was described and compared across *Leishmania* spp. by Fraga et al. [15]; this target allows for the discrimination between all clinically relevant *Leishmania* species from the Old and New World in human clinical specimens [10].

## 2. Materials and Methods

### 2.1. Study Area

RER constitutes the study area; it is located in northeastern Italy and covers 22,445 km^2^. The region has 4,438,937 inhabitants. The territory is constituted by the Apennine Mountains (25% of the territory), hills (27%), and an alluvial plain (48%).

### 2.2. Study Design and Sample Collection

A total of 109 samples that were collected between 1 January 2014 and 31 December 2020 were included in the study; specimens were 10 μm-wide sections from the formalin-fixed and paraffin-embedded (FFPE) biopsies of mucosal or skin tissues obtained from 104 patients with confirmed TL. The samples were obtained within the diagnostic and surveillance activities of the Regional Reference Laboratory for leishmaniasis (RRL) at the Unit of Microbiology, University Hospital of Bologna, Italy; FFPE samples from suspected cases of TL were sent to RRL from different provinces within RER for molecular confirmation of *Leishmania* infection. Samples from patients with confirmed TL were stored at −20 °C until the molecular analyses were performed. The demographic and clinical data of TL cases were collected within the Skin_Leish_RER network, an informal group including 10 diagnostic units that was recently established to improve the surveillance of TL cases in RER [5]. Data were organized in an electronic database that was shared with network members by using a password-protected platform. The data of the patients enrolled in this study were anonymized by an alphanumerical code. This study was conducted in accordance with the Declaration of Helsinki, and the protocol was approved by the Ethics Committee of the Area Vasta Emilia Centro (study number: n° EM414-2023_97/2017/O/Tess/AOUBo).

### 2.3. Case Definition and Characterization

A TL case was identified based on the WHO definition [1,7], i.e., a patient with suggestive cutaneous and/or mucosal lesion/s, in which *Leishmania* parasites were detected by histology and/or by molecular methods. All TL cases included in the study were confirmed by real-time PCR as described [16]. Different clinical forms of TL were classified following the designations that were established by the European LeishMan consortium [7]; CL refers to the presence of skin lesion(s), and ML refers to the presence of mucosal lesion(s) without skin involvement [1]. A case was considered potentially imported when a history of travel abroad in countries that are endemic for leishmaniasis was reported in the 12 months before the lesions’ onset. If no travel history in leishmaniasis endemic regions was reported in the previous 12 months, the case was considered autochthonous.

### 2.4. DNA Extraction, Amplification, and Sequencing

DNA from the FFPE biopsies was extracted by the NucleoSpin^®^ DNA FFPE XS kit (Macherey-Nagel, Duren, Germany) or by the Maxwell “RSC/CSC Decktray” kit Maxwell^®^ CSC DNA FFPE Kit (Promega, Madison, WI, USA) according to the manufacturer’s instructions. Confirmation of *Leishmania* DNA presence in extracted samples was carried out by an in-house real-time PCR assay targeting regions of the minicircle kinetoplast (k)DNA [16] and of the small-subunit rRNA gene, respectively.

The ITS1 fragment of 320 bp was amplified in a PCR final volume of 30 µL using the primer pairs LITSR/L5.8S and the mastermix GoTaq^®^ G2 Hot Start Colorless (Promega, Madison, WI, USA), according to the amplification conditions described in [17], with some modifications: 95 °C for 2 min, followed by 35 cycles at 94 °C for 30 s, 50 °C for 30 s, and 72 °C for 1 min, with a final extension at 72 °C for 5 min. In the case of negative results, a second round of ITS1 amplification was performed using the same primer set as above. To check the absence of carryover contamination a no template control (NTC) was run alongside samples in every amplification reaction and NTC from the amplification round 1 was used in round 2 as a template.

The amplification of the hsp70 coding sequence fragment was optimized for its application on FFPE sections and was performed using primers to amplify the N-, P-, and Ps-fragment (Table 1) in a three-step nested PCR (Figure S1). Amplification of the N-fragment was previously reported [18], while smaller P and Ps primer sets were designed to increase the PCR sensitivity as FFPE samples often contain damaged nucleic acid and a scarce amount of parasitic DNA. The Ps smaller amplicons include known variable sites in their sequence, distinguishing among *L. infantum*, *L. donovani*, *L. major*, and *L. tropica*. The PCR reaction was performed by the Thermocycler T-Gradient ThermoBlock (Biometra, Göttingen, Germany) using the Hotstartaq plus kit mix (Qiagen, Hilden, Germany) according to the manufacturer’s instructions and amplification conditions as described [18]. In case no evident amplification for the N-fragment (593 bp) was obtained, the N-fragment PCR reaction was purified with Exosap^®^ (Thermofisher, Waltham, MA, USA) following the manufacturer’s instructions and used as a template for a nested PCR reaction set up with primers for the P-fragment (Table 1; Figure S1) to obtain a smaller amplicon of 295 bp. If still no amplification was obtained, the P-fragment PCR reaction was purified with Exosap^®^ and used as a template for the amplification of the inner “Ps-fragment” of 262 bp. To check the absence of carryover contamination in the double nested hsp70 amplification, an NTC was run alongside samples in every amplification reaction, and the NTC from the N-fragment amplification was used as a template for the P-fragment PCR reaction. Subsequently, the same procedure was performed for Ps-fragment amplification.

For sequencing, both the ITS1 and hsp70 amplicons (N-, P-, or Ps-fragment) were purified with the Agencourt AMPure^XP^ PCR Purification Kit (Beckman Coulter™, Indianapolis, IN, USA). Sanger sequencing reactions were carried in both directions using the GenomeLab DTCS Quick Start Kit (Beckman Coulter) in 20 µL of final volume according to the manufacturer’s instructions. The primers used for sequencing were the same used in the amplification reactions (Table 1; Figure S1). Sequencing was performed with a Sciex GenomeLab^TM^ GeXP sequencer (Beckman Coulter).

### 2.5. Whole Genome Sequenced (WGS)-Based Analysis of hsp70 Gene

Next generation sequencing (NGS) reads of 149 *L. donovani* complex isolates were obtained from Franssen et al. [19]. The sequence reads were trimmed with Trimmomatic (https://github.com/usadellab/Trimmomatic (accessed on 16 November 2023)), including the removal of paired-end adaptors using the options: ‘ILLUMINACLIP:TruSeq3-PE.fa:2:30:10 TRAILING:25 MINLEN:60′. The trimmed reads were mapped against the genomic DNA of the reference strain, JPCM5 (MCAN/ES/98/LLM-724) of *L. infantum* (TriTrypDB v65 (https://tritrypdb.org (accessed on 16 November 2023)), with BWA v0.7.17 (https://github.com/lh3/bwa (accessed on 16 November 2023)) and using the bwa-mem aligner and default options. Single nucleotide polymorphisms (SNP) and InDel variants were called using BCFtools v1.16 utilities (https://github.com/samtools/bcftools (accessed on 16 November 2023)). The first vcf files were generated with bcftools mpileup and then variants were called with bcftools call and InDels were normalized with bcftools norm (default options). A reference-based assembly with bcftools consensus and the *-H I* option (use IUPAC code for all genotypes) was performed using all detected variants in the vcf format to the JPCM5 LINF_280036000 hsp70 coding sequence (LinJ.28:1112868-1114832, TriTrypDB v65).

### 2.6. Data Analysis

The sequence results of hsp70 and ITS1 were analyzed and assembled using the “Sequencing” and “Investigator” packages of the GenomeLab^TM^ System software, version 11.0.24. The consensus sequences obtained were identified as the *Leishmania* spp. species by a Blast search in Gen Bank at NCBI. When necessary, hsp70 sequences were cut to the same size including the 218 bp of the Ps fragment before the alignment. For all the samples successfully typed with both methods, the discriminatory power was evaluated by the Simpson’s index of diversity using a free online tool (http://www.comparingpartitions.info/index.php?link=Tool (accessed on 15 November 2023)).

## 3. Results

Molecular typing was carried out in 109 sections of FFPE biopsies that were collected from TL lesions; amplification and sequencing were successful for at least one target (ITS1 and/or hsp70) in 88 samples (80.7%) (Table S1). The 88 sequenced samples were collected from 83 patients (n = 77 CL cases and n = 6 ML cases); 2 samples were obtained from 1 patient with multiple lesions and 8 samples from 4 patients undergoing relapse. In all five cases, the typing results of samples obtained from the same patient were concordant. Among the 88 typed samples, 79 samples were obtained from 75 patients that were considered autochthonous cases and 9 samples were from 8 cases, for whom the place of infection could be outside Italy, as they had a recent travel history to countries that were endemic for leishmaniasis.

A total of 77 samples out of 109 (70.6%) were successfully typed for ITS1 (Accession Number, A.N., reported in Table S1); they were obtained from 73 TL cases, as 8 samples were obtained from 4 patients exhibiting relapses or multiple lesions (3 from *L. infantum* and 1 from *L. major*). As shown in Table 2, 67 TL cases (91.8%) were identified as *L. infantum*, 4 (5.5%) as *L. major*, and 2 (2.7%) as *L. tropica*. All samples typed by ITS1 as *L. infantum* were previously classified as autochthonous, while the six patients, whose lesions were typed as *L. major* and *L. tropica*, reported traveling to Tunisia (n = 3, *L. major*), Burkina Faso (n = 1, *L. major*), or Morocco (n = 2, *L. tropica*).

The hsp70 fragment was successfully typed in 87 samples (from 83 TL cases) out of 109 (79.8%) (see A.N. in Table S1). For all 87 specimens, a consensus sequence of at least 218 bp was obtained.

A total of 76 out of 83 patients (91.6%) were grouped within the *L. donovani* complex; in this group, we observed 8 sequence variants with respect to the sequence of the reference strain *L. infantum* JPCM5 (A.N.: XM_001470287.1_inf_LLM-877), named from A to H, while 5 cases (6.0%) were typed as *L. major*, and 2 (2.4%) as *L. tropica* (Table 2). By comparing the typing results for samples that could be amplified by both PCRs (n = 76), we observed that ITS1 typing exhibited a lower discrimination power as compared to hsp70, with a Simpson’s index of diversity of 0.173 (95% CI: 0.060–0.286) and 0.731 (95% CI: 0.665–0.796), respectively (*p*-value < 0.001).

All cases typed within the *L. donovani* complex (n = 76) were previously classified as autochthonous, except for one case exhibiting sequence variant B that reported a travel history to Bangladesh. The hsp70 sequencing allowed us to identify an additional *L. major* case in a sample that was not amplified by ITS1 PCR; this case reported a travel history to the Middle East.

**Figure 1:**
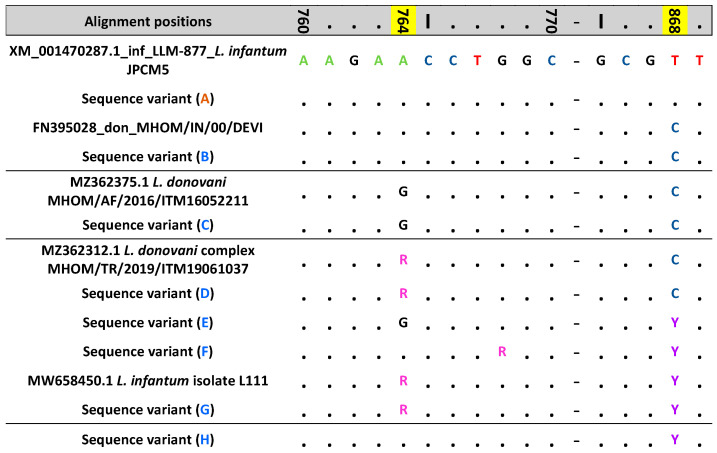
Representation of the partial sequences of the hsp70 sequence variants obtained in the present study. The hsp70 partial sequences of the variants, obtained from the 75 Italian autochthonous cases of TL collected in 2014–2020 were aligned with sequences available on NCBI Genbank by Clustal W, as implemented in BioEdit v.7.0.8.0 [21]. Highlighted in yellow, the nucleotide positions 764 and/or 868 exhibiting differences in the variants B–H with respect to the JPCM5 reference strain. Nucleotides were indicated by different colors: A, green; G, black; C, navy blue, T, red; R, pink; Y, purple. Sequence variant A, identical to the JPCM5 reference strain, is depicted in orange while variants B-H are depicted in blue.

The eight hsp70 sequence variants that were observed within the *L. donovani* complex typed cases were further investigated (Figure 1). The sequence of hsp70 variant A was detected in n = 29 autochthonous cases (38.7% of total autochthonous cases within the *L. donovani* complex); this sequence was identical to the reference *L. infantum* JPCM5 strain (A.N.: XM_001470287.1_inf_LLM-877) (Table S2). For sequence variants B-E (n = 15 autochthonous cases, 20.0%, and 1 imported case) and F-H (n = 31 cases, 41.3%) differences in the nucleotide sequences with respect to the JPCM5 reference strain were observed only in positions 764 and/or 868, both within the Ps fragment. In position 764, we observed the A → G substitution in C and E sequence variants or the presence of the corresponding degenerated base R if both nucleotides were present in the same position, as for sequence variants D and G. The T → C substitution was observed in position 868 for sequence variants B-D, and the corresponding degenerated nucleotide Y in E-H. In addition, in sequence variant F, which was detected in one sample, a degenerated nucleotide R was observed in position 768.

A BLAST search of the B and C sequence variants returned 100% identity with both *L. donovani* (B 63/66; C 3/3) and *L. infantum* (B 3/66) strains, although the former was more represented (Table S2). On the other hand, for the sequence variants D and G (Figure 1), which present one or both degenerate sites, we found in GenBank an identical sequence of *L. donovani* complex from Turkey (MHOM/TR/2019/ITM19061037) and *L. infantum* of unknown place of infection (L111, A.N.:MW658450.1), respectively, included in a previous work [20]. The sequence variants E, F, and H have not yet been reported.

A total of 67 out of the 76 cases (88.2%) that were typed as *L. donovani* complex by the hsp70 sequencing were identified as *L. infantum* with ITS1, while the remaining 11 cases could not be typed with ITS1. The ITS1 and hsp70 typing results overlapped for *L. major* (n = 4 cases) and *L. tropica* (n = 2 cases), nevertheless one sample identified as *L. major* by hsp70 could not be typed with ITS1 (Table 2).

To explore the global hsp70 variability within the *L. donovani* complex, we built the hsp70 coding sequences by using the whole genome sequence reads from 149 *L. donovani* complex strains isolated in different countries, which were available from Fransen et al. [19] (Table S3). The alignment of these reads to the reference genome JPCM5 in the N, P, or Ps fragments of the hsp70 gene showed no variations for the *L. infantum* strains and one single polymorphic site for the *L. donovani* strains. Furthermore, by focusing on the polymorphic sites of the sequence variants B-H of this study, we observed that, in position 764, all reads exhibited an A among all 149 strains, while regarding position 868, all strains identified as *L. infantum* showed a T, and the ones identified as *L. donovani* showed a C; in both polymorphic sites, no degenerated base was observed (coverage ranged from 50 to 200X, approximately).

## 4. Discussion

The molecular surveillance of circulating *Leishmania* parasites in endemic regions is important for several reasons. Clinicians may not always be able to distinguish between autochthonous and imported cases of TL, which may necessitate different treatment regimens [3,22]. Moreover, new *Leishmania* species/strains may be introduced and transmitted by local sand flies in endemic areas with unpredictable consequences; in southern Europe, particular attention should be paid to the potential establishment of imported anthroponotic species, such as *L. tropica* and *L. donovani*, while the risk of introduction of new zoonotic *Leishmania* species is considered low [23].

Several molecular approaches for the identification of *Leishmania* species have been described and exhibit different discriminatory power [3]. In this study, specimens obtained from the skin and mucosal biopsies from 83 TL cases that were diagnosed in northeastern Italy between 2014 and 2020 were amplified and sequenced by employing two typing tools, i.e., ITS1 and hsp70; the combination of these two markers as well as the improvement of PCR tools for hsp70 amplification allowed us to obtain species identification in 80% of the FFPE samples.

The typing results of ITS1 and hsp70 showed a good agreement in the detection of imported cases of TL by identifying four and two cases caused by *L. major* and *L. tropica*, respectively, with one additional *L. major* case detected by hsp70 only. Our findings are in line with previous reports regarding the geographic species distribution of imported leishmaniasis into Europe; *L. major* and *L. tropica* are well-known causes of CL in North Africa and the Middle East [24] and *L. major* circulation has been observed in Burkina Faso [25]. Among the potential imported cases, we also identified the *L. donovani* complex by hsp70 in a patient with CL who recently traveled to Bangladesh. CL cases caused by *L. donovani* have been identified in South Asia, namely Sri Lanka [26], with no reports from Bangladesh so far. Nevertheless, an autochthonous origin cannot be excluded from this case.

We observed that all the autochthonous TL cases belonged to the *L. donovani* complex; furthermore, ITS1 sequences were homogeneous and completely overlapping with *L. infantum* reference strains [27], while the hsp70 sequences obtained from the same samples showed a high degree of variability. In addition to less represented sequence variants, autochthonous cases were mainly segregated into two groups corresponding to variants A and G, with the former overlapping with the sequence of *L. infantum* reference strains from the Mediterranean basin, while variant G has not been previously reported from the same area.

The variability observed among strains circulating in a relatively small geographic area was unexpected, especially if we consider that *L. infantum* is the only autochthonous species in Italy. Indeed, high conservation was observed when the hsp70 Ps sequence of 149 *L. donovani* complex genomes from different geographic regions, including Europe, North Africa (only two sequences), the Middle East, Central, Eastern and Southern Asia, East Africa, and Latin America [19], was explored in silico. At position 868, a single polymorphic site that clearly discriminates the species within the *L. donovani* complex was observed. This specifically presents a T for all *L. infantum* strains, as we found for sequence variant A, and a C for all *L. donovani* strains, and as we found for sequence variants B, C, and D (Table S3), supporting BLAST results. Notably, none of the hsp70 sequences from the in silico analysis evidenced the presence of a G at position 764, as it was in our sequence variants C and E, and the corresponding degenerated nucleotide R as in sequence variants D and G.

We observed that among eight autochthonous TL cases which were identified as B, C, and D sequence variants, thus showing identity with *L. donovani* strains, four were typed as *L. infantum* by ITS1. Our results are in line with the observation that *L. infantum* and *L. donovani* species form a continuum and are sometimes intermingled, probably as a consequence of progressive genetic differentiation [28]. In addition, the possibility of past hybridization events between *L. infantum* and *L. donovani* in the Italian strains could not be ruled out as a cause of discordant or ambiguous results based on single marker approaches.

In line with this hypothesis, multi-locus microsatellite typing detected a peculiar strain causing VL in northeastern Italy that was highly divergent from the *L. infantum* population commonly causing leishmaniasis in humans and dogs in the Mediterranean basin [29]; whole genome sequencing revealed a common origin of this *Leishmania* population with the *L. infantum*/*L. donovani* putative hybrids from Cyprus [30]. Therefore, the typing results obtained in autochthonous cases of TL in the present study likely reflect a high variability within the *L. donovani* complex strains.

The hsp70 proteins form a highly conserved family within the *L. donovani* complex [15]. However, we found three mutations within the Ps fragment in the autochthonous cases of TL, including two silent mutations (768, 868) and one mutation (764) that involves an amino acid replacement. A parallel study of the *Leishmania* population structure is ongoing in the selected area, including hsp70 typing in viscerotropic strains, and could be useful in exploring the correlation between genotypic and phenotypic features and further evaluating the potential application of the hsp70 Ps fragment as an epidemiological and/or prognostic marker.

This study incurred some limitations. Firstly, we collected strains from a limited geographic area; thus, the results cannot give information about the dermotropic *Leishmania* strains circulating in the entire Italian peninsula. Further, only two genetic markers were typed by Sanger sequencing; a multi-locus or a genome-wide approach would contribute to better clarifying the characteristics of the diverse *Leishmania* parasites that cause TL in northeastern Italy.

## 5. Conclusions

The combined analysis of ITS1 and hsp70 markers confirmed the usefulness of molecular tools for the species identification of *Leishmania* directly in FFPE samples from TL cases and for correctly identifying exotic dermotropic species. We observed that dermotropic strains circulating in northeastern Italy belong to the *L. donovani* complex and exhibit several variants in the hsp70 sequence. High genomic plasticity and adaptive capacity in *Leishmania* spp. suggest the need to develop easy-to-use tools to integrate molecular surveillance in leishmaniasis control programs [31]. Within this strategy, the validation of hsp70 single nucleotide polymorphisms should be implemented on additional *L. infantum* dermotropic strains from southern Europe.

## Figures and Tables

**Table 1:** hsp70 primers used for the amplification and sequencing of hsp70 fragments.

hsp70 Fragments	Primer Name	Primer Sequence	Position *	Amplicon Size
**N-fragment**	F25_var (Sense)	5′_GGACGCCGGCACGATT**G**CT_3′	480–498	593 bp
	R617_var (Antisense)	5′_CGAAGAAGTCCGA**C**ACGAGGGA_3′	1072–1051	
**P-fragment**	F-paraf (Sense)	5′_GGACTTCGACAACCGCCTC_3′	699–717	295 bp
	R-paraf (antisense)	5′_CTTGTCCATCTTCGCGTCCT_3′	993–974	
**Ps-fragment**	For-paraf-s (forward)	5′_CGTCACGTTCTTCACCGAGGAGT_3′	717–739	262 bp
	Rev-paraf-s (reverse)	5′_GTCCTGCAGCACGCGCTCCAC_3′	978–958	

* Annealing position in GenBank accession number: XM_001470287.1_inf_LLM-877_*Leishmania infantum* JPCM5, according to [18]. N-fragment primers set were designed according to [18], or with modification (in bold). The sets of primers in the grey-shaded rows are new primers, designed using the Primer Express^®^ software v.3.0 (Applied Biosystems, Foster City, CA, USA). In silico analytic specificity was evaluated on a panel of *Leishmania* and other Trypanosomatidae hsp70 sequences available from GenBank.

**Table 2:** Comparison of the ITS1 and hsp70 typing results (n = 83 cases).

		hsp70											
		*L. donovani* Complex								*L. major*	*L. tropica*	NA	Total
		Sequence Variant											
		A	B	C	D	E	F	G	H				
**ITS1**	***L. infantum***	25	1	4	1	6	1	28	1	0	0	0	67
	***L. major***	0	0	0	0	0	0	0	0	4(4)	0	0	4(4)
	***L. tropica***	0	0	0	0	0	0	0	0	0	2(2)	0	2(2)
	**NA**	4	2(1)	1	0	1	0	1	0	1(1)	0	0	10(2)
	**Total**	**29**	**3(1)**	**5**	**1**	**7**	**1**	**29**	**1**	**5(5)**	**2(2)**	**0**	**83(8)**

NA; not amplified. In brackets; the number of imported cases.

## Data Availability

The sequences reported in the present paper were deposited in the GenBank database: Accession Numbers can be found in Table S1.

## Supplementary Materials

The following supporting information can be downloaded at: https://www.mdpi.com/article/10.3390/pathogens13010019/s1, Figure S1: PCR reactions and sequencing scheme; Table S1: Metadata on the 88 *Leishmania* spp. samples sequenced in this study; Table S2: Results of the BLAST search of hsp70 sequences for variants A, B, C.; and Table S3: Samples used in the WGS analysis.

## References

[1] Gradoni L., López-Vélez R., Mokni M. (2017). Manual on Case Management and Surveillance of the Leishmaniases in the WHO European Region.

[2] Ruiz-Postigo J.A., Jain S., Madjou S., Maia-Elkhoury A.N., Valadas S., Warusavithana S., Osman M., Yajim A., Lin A., Beshah A. (2022). Global Leishmaniasis Surveillance: 2021, Assessing the Impact of the COVID-19 Pandemic.

[3] Van der Auwera G., Dujardin J.C. (2015). Species typing in dermal leishmaniasis. Clin. Microbiol. Rev..

[4] Maroli M., Rossi L., Baldelli R., Capelli G., Ferroglio E., Genchi C., Gramiccia M., Mortarino M., Pietrobelli M., Gradoni L. (2008). The northward spread of leishmaniasis in Italy: Evidence from retrospective and ongoing studies on the canine reservoir and phlebotomine vectors. Trop. Med. Int. Health.

[5] Gaspari V., Gritti T., Ortalli M., Santi A., Galletti G., Rossi A., Rugna G., Mattivi A., Matteo G., Belloli G.L. (2022). Tegumentary Leishmaniasis in Northeastern Italy from 2017 to 2020: A Neglected Public Health Issue. Int. J. Environ. Res. Public Health.

[6] Mattivi A., Massimiliani E., Cagarelli R., Albieri A. Leishmaniosi in Emilia-Romagna, Aggiornamento Epidemiologico 1999–2015. https://salute.regione.emilia-romagna.it/normativa-e-documentazione/rapporti/malattie-infettive/leishmaniosi-er-epidemiologia-1999-2015.

[7] Guery R., Walker S.L., Harms G., Neumayr A., Van Thiel P., Gangneux J.P., Clerinx J., Söbirk S.K., Visser L., Lachaud L. (2021). Clinical diversity and treatment results in Tegumentary Leishmaniasis: A European clinical report in 459 patients. PLoS Negl. Trop. Dis..

[8] Gaspari V., Zaghi I., Macrì G., Patrizi A., Salfi N., Locatelli F., Carra E., Re M.C., Varani S. (2020). Autochthonous Cases of Mucosal Leishmaniasis in Northeastern Italy: Clinical Management and Novel Treatment Approaches. Microorganisms.

[9] Faucher B., Pomares C., Fourcade S., Benyamine A., Marty P., Pratlong L., Faraut F., Mary C., Piarroux R., Dedet J.P. (2011). Mucosal Leishmania infantum leishmaniasis: Specific pattern in a multicentre survey and historical cases. J. Infect..

[10] Van der Auwera G., Bart A., Chicharro C., Cortes S., Davidsson L., Di Muccio T., Dujardin J.C., Felger I., Paglia M.G., Grimm F. (2016). Comparison of Leishmania typing results obtained from 16 European clinical laboratories in 2014. Euro Surveill..

[11] Chicharro C., Llanes-Acevedo I.P., García E., Nieto J., Moreno J., Cruz I. (2013). Molecular typing of Leishmania infantum isolates from a leishmaniasis outbreak in Madrid, Spain, 2009 to 2012. Euro Surveill..

[12] Van der Auwera G., Ravel C., Verweij J.J., Bart A., Schönian G., Felger I. (2014). Evaluation of four single-locus markers for Leishmania species discrimination by sequencing. J. Clin. Microbiol..

[13] Schönian G., Kuhls K., Mauricio I.L. (2011). Molecular approaches for a better understanding of the epidemiology and population genetics of Leishmania. Parasitology.

[14] Folgueira C., Cañavate C., Chicharro C., Requena J.M. (2007). Genomic organization and expression of the HSP70 locus in New and Old World Leishmania species. Parasitology.

[15] Fraga J., Montalvo A.M., De Doncker S., Dujardin J.C., Van der Auwera G. (2010). Phylogeny of Leishmania species based on the heat-shock protein 70 gene. Infect. Genet. Evol..

[16] Gaspari V., Ortalli M., Foschini M.P., Baldovini C., Lanzoni A., Cagarelli R., Gaibani P., Rossini G., Vocale C., Tigani R. (2017). New evidence of cutaneous leishmaniasis in north-eastern Italy. J. Eur. Acad. Dermatol. Venereol..

[17] El Tai N.O., El Fari M., Mauricio I., Miles M.A., Oskam L., El Safi S.H., Presber W.H., Schönian G. (2001). Leishmania donovani: Intraspecific polymorphisms of Sudanese isolates revealed by PCR-based analyses and DNA sequencing. Exp. Parasitol..

[18] Van der Auwera G., Maes I., De Doncker S., Ravel C., Cnops L., Van Esbroeck M., Van Gompel A., Clerinx J., Dujardin J.C. (2013). Heat-shock protein 70 gene sequencing for Leishmania species typing in European tropical infectious disease clinics. Euro Surveill..

[19] Franssen S.U., Durrant C., Stark O., Moser B., Downing T., Imamura H., Dujardin J.C., Sanders M.J., Mauricio I., Miles M.A. (2020). Global genome diversity of the Leishmania donovani complex. Elife.

[20] Van der Auwera G., Davidsson L., Buffet P., Ruf M.T., Gramiccia M., Varani S., Chicharro C., Bart A., Harms G., Chiodini P.L. (2022). Surveillance of leishmaniasis cases from 15 European centres, 2014 to 2019: A retrospective analysis. Euro Surveill..

[21] Hall T.A. (1999). BioEdit: A User-Friendly Biological Sequence Alignment Editor and Analysis Program for Windows 95/98/NT. Nucleic Acid Symposium Series.

[22] de Vries H.J.C., Schallig H.D. (2022). Cutaneous Leishmaniasis: A 2022 Updated Narrative Review into Diagnosis and Management Developments. Am. J. Clin. Dermatol..

[23] Di Muccio T., Scalone A., Bruno A., Marangi M., Grande R., Armignacco O., Gradoni L., Gramiccia M. (2015). Epidemiology of Imported Leishmaniasis in Italy: Implications for a European Endemic Country. PLoS ONE.

[24] Hotez P.J., Savioli L., Fenwick A. (2012). Neglected tropical diseases of the Middle East and North Africa: Review of their prevalence, distribution, and opportunities for control. PLoS Negl. Trop. Dis..

[25] Konate I., Sangare I., Zoungrana J., Meda Z.C., Kafando C., Sawadogo Y., Dabiré R., Meda N., Diallo B., Andonaba J.B. (2020). Description of a new epidemic focus of cutaneous Leishmaniasis major in western Burkina Faso. Pan. Afr. Med. J..

[26] Katakura K. (2009). Molecular epidemiology of leishmaniasis in Asia (focus on cutaneous infections). Curr. Opin. Infect. Dis..

[27] Kuhls K., Mauricio I.L., Pratlong F., Presber W., Schönian G. (2005). Analysis of ribosomal DNA internal transcribed spacer sequences of the Leishmania donovani complex. Microbes Infect..

[28] El Baidouri F., Diancourt L., Berry V., Chevenet F., Pratlong F., Marty P., Ravel C. (2013). Genetic structure and evolution of the Leishmania genus in Africa and Eurasia: What does MLSA tell us. PLoS Negl. Trop. Dis..

[29] Rugna G., Carra E., Bergamini F., Calzolari M., Salvatore D., Corpus F., Gennari W., Baldelli R., Fabbi M., Natalini S. (2018). Multilocus microsatellite typing (MLMT) reveals host-related population structure in Leishmania infantum from northeastern Italy. PLoS Negl. Trop. Dis..

[30] Bruno F., Castelli G., Li B., Reale S., Carra E., Vitale F., Scibetta S., Calzolari M., Varani S., Ortalli M. Genomic and epidemiological evidence for the emergence of a putative *L. donovani*/*L. infantum*; hybrid with unusual epidemiology in Northern Italy. https://ssrn.com/abstract=4611485.

[31] Domagalska M.A., Dujardin J.C. (2020). Next-Generation Molecular Surveillance of TriTryp Diseases. Trends Parasitol..

