# Supplementary material for: Molecular Typing of Leishmania spp. Causing Tegumentary Leishmaniasis in Northeastern Italy, 2014–2020
Source: Pathogens. 2023 Dec 24;13(1):19. doi: 10.3390/pathogens13010019 (PMC10820635; doi:10.3390/pathogens13010019)
Supplement: Supplementary file 1 [file pathogens-13-00019-s001.zip › Figure S1.pdf]

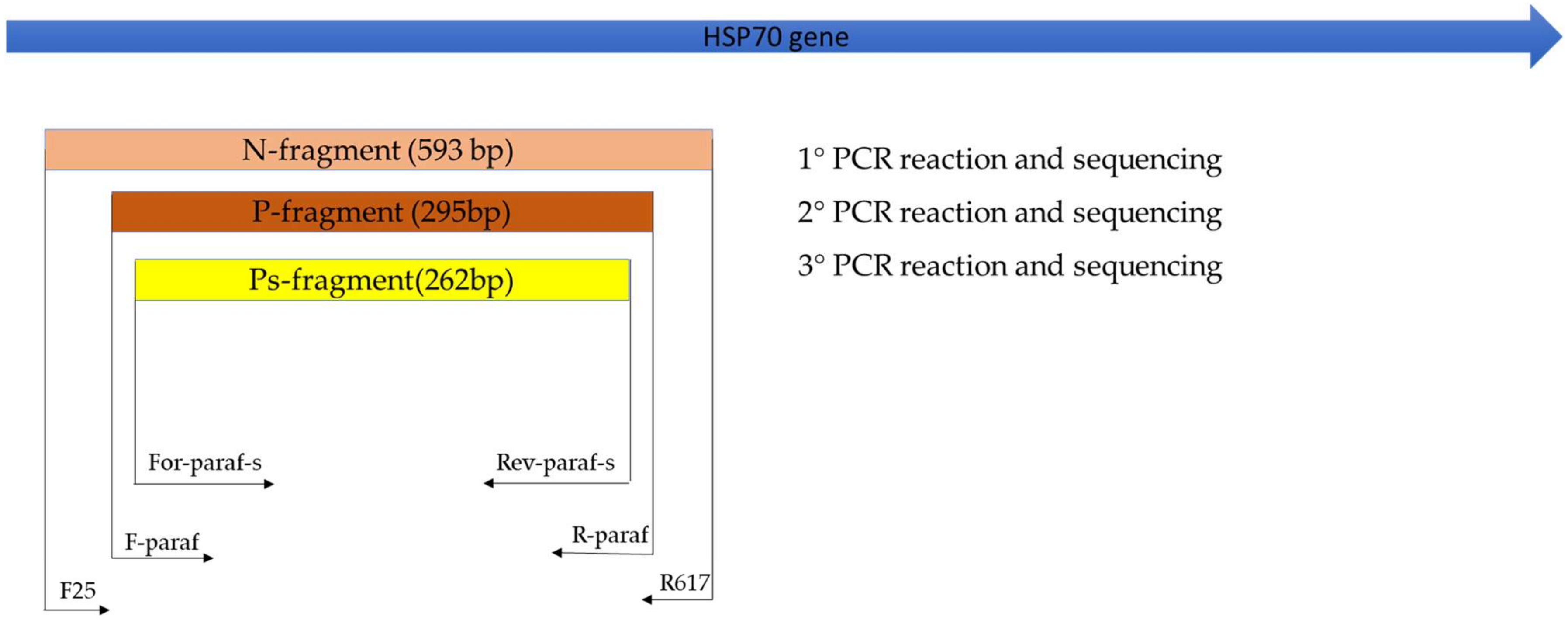

Figure S1. Primers used and amplification products obtained and sequenced within the hsp70 coding region.
